# Notes and News

**Published:** 1896-02-22

**Authors:** 


### Notes and News.

(Collected and Communicated.)

HOSPITAL MEETINGS.

The Annual Meeting of the Governors of the West London Hospital, Hammersmith Road, W., will take place in the Board Room on Monday, March 9th, at 4.30 p.m.

The Annual General Court of the Governors of the City of London Hospital for Diseases of the Chest, Victoria Park, E , will be held at the hospital on Thursday, February 27th, at 4 p.m.

The Annual General Board of Governors of the St. John's Hospital for Diseases of the Skin, 49, Leicester Square, W.C., will be held on Friday, February 21st, at 3 p.m., at the Hotel Victoria, Northumberland Avenue.

The Annual Court of the Seamen's Hospital Society will be held in the Mansion House on Friday, the 28th inst., at twelve o'clock.

A Festival Dinner in aid of the Annual Fund of the Cabdrivers' Benevolent Association will be held on Wednesday, March 11th, at the Hotel Metropole, under the presidency of H.R.H. the Duke of York.

H.R.H. the Prince of Wales has accepted the office of vice-patron to the Metropolitan Hospital Sunday Fund.

The Duchess of Albany paid an informal visit to the wards of the National Hospital for the Paralysed and Epileptic on Tuesday.

The annual general court of the Charing Cross Hospital was held in the board-room of the hospital on the 19th inst. at two p.m.

The quarterly court of the Hospital for Consumption and Diseases of the Chest will be held in the board-room of the hospital on Thursday, February 27th, at a quarter to five p.m.

Through the will of the late Mr. David Guillan, the Dundee Royal Infirmary has benefited to the extent of nearly £16,000. The bequest, it is understood, is unconditional, and it is proposed that the money should be invested, and the interest applied to revenue.

The following gentlemen have been successful in obtaining commissions in the Medical Staff of Her Majesty's army at the recent examination in London:—E. T. F. Birrell, A. H. Morris, S. A. Archer, E. W. W. Cochrane, M. Swabey, A. J. MacDougall, G. B. Riddick, H. Hewetson, R. W. Clements. The names are given in the order of merit.

The Secretary of the British Home for Incurables has received £2 11s. 6d. from H.R.H. the Princess of Wales (patroness of the charity), being a further portion of part proceeds of the sale of Canon Fleming's sermon, "Recognition in Eternity," thus making a total of £696 11s. received from that source; also £21 from the Leathersellers' Company, £21 from the Clothworkers' Company (annual), and £3 3s. from the Dyers' Company. Candidates either for the Home or the Pension must make application without delay, as the list for the May Election will be closed on March 12th next.

There are few tasks attended with more difficulties and disagreeables than the erection of isolation hospitals. Local prejudice is always hard to surmount, and the amount of opposition raised is always great. The height to which this feeling can rise was illustrated at Oakbridge, near Stroud, last month, when a mob surrounded a recently-erected small-pox hospital and burnt it to the ground. An even more astonishing scene took place on the 29th ultimo, when a mob surrounded an unfortunate small-pox patient who was being conveyed to a cottage purchased by the Joint Hospital Board for isolating cases of disease. The ambulance which contained the sufferer was escorted by two sanitary inspectors, but when they drew near the cottage, some six miles from Stroud, it is reported by the local press, that about a hundred persons, many of them armed with sticks, surrounded the party, threw stones at the ambulance, and, turning the horses round, forced them to return to the town. The patient was replaced in the common lodging-house whence he had been taken, and summonses have been taken out against obstructionists whose conduct appears to have been inhuman and irrational. The ringleaders, in both cases, were arrested, but no punishment they may receive will restore the wasted money, nor, probably, quell the opposition to the scheme.

The report of the proceedings at the Quarterly Court of the Governors of the Radcliffe Infirmary, Oxford, in the *Oxford Chronicle*, of the 25th ult., must cause the most enthusiastic supporter of that institution amazement and justifiable despair. We have pointed out more than once during the past year that, although it was decided by the governors to request the committee to put their house in order by taking steps to appoint a superintendent, not only have no such steps apparently been taken, but the reference to the committee is entirely ignored. It would appear that the institution is managed in defiance of all ordinary business principles. We qualified as governors of the Radcliffe Infirmary in the hope that we might have a notice sent us of the meetings of the governors, and so be enabled to take some part in the administration of the Radcliffe Infirmary. But no notice of any meeting has been sent, though we know that meetings have been held. This omission to issue proper notices to the Governors is alone sufficient to account for the lack of interest and the small attendance at the Quarterly Courts. There must be some men of business in Oxford, and it is matter for regret everywhere, to judge by the proceedings at the Quarterly Courts, that the best of them, at any rate, seem to shun the infirmary as they would avoid the plague. Possibly this absence of business men is due to a feeling on their part that it is a waste of time under present circumstances to endeavour to infuse energy and administrative spirit into the conduct of the affairs of this most unfortunate institution. A modicum of energy, and the introduction of ordinary business methods would not only place the Radcliffe Infirmary in funds, but awaken a genuine interest in its affairs, which proceedings like those at the last Quarterly Court cannot fail to render difficult, if not impossible.

